# Supplementary figures and images for: High-Content Screening and Computational Prediction Reveal Viral Genes That Suppress the Innate Immune Response
Source: mSystems. 2022 Mar 23;7(2):e01466-21. doi: 10.1128/msystems.01466-21 (PMC9040872; doi:10.1128/msystems.01466-21)

Figure S1

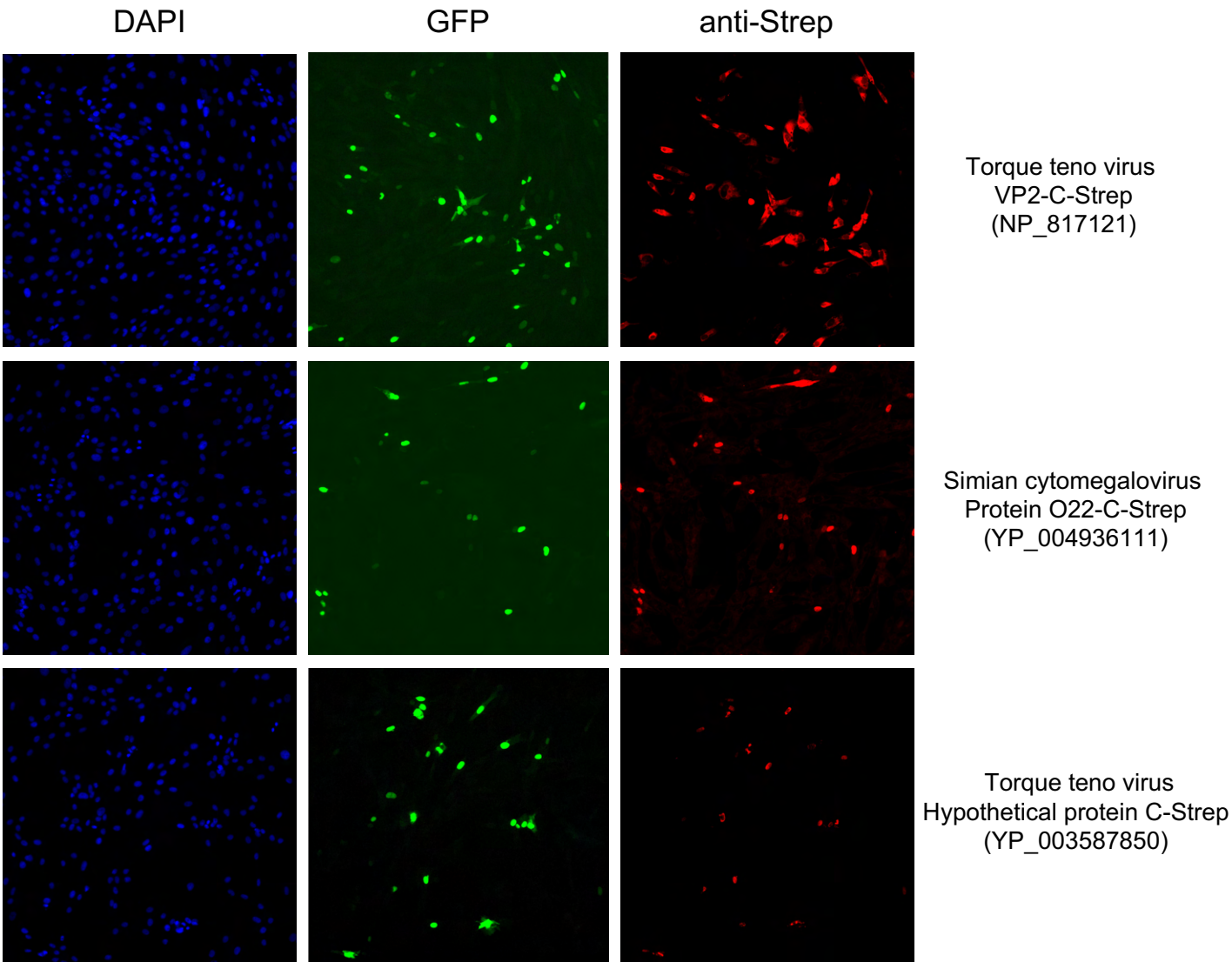

Supplement: FIG S1 [file msystems.01466-21-sf001.pdf]

Figure S2

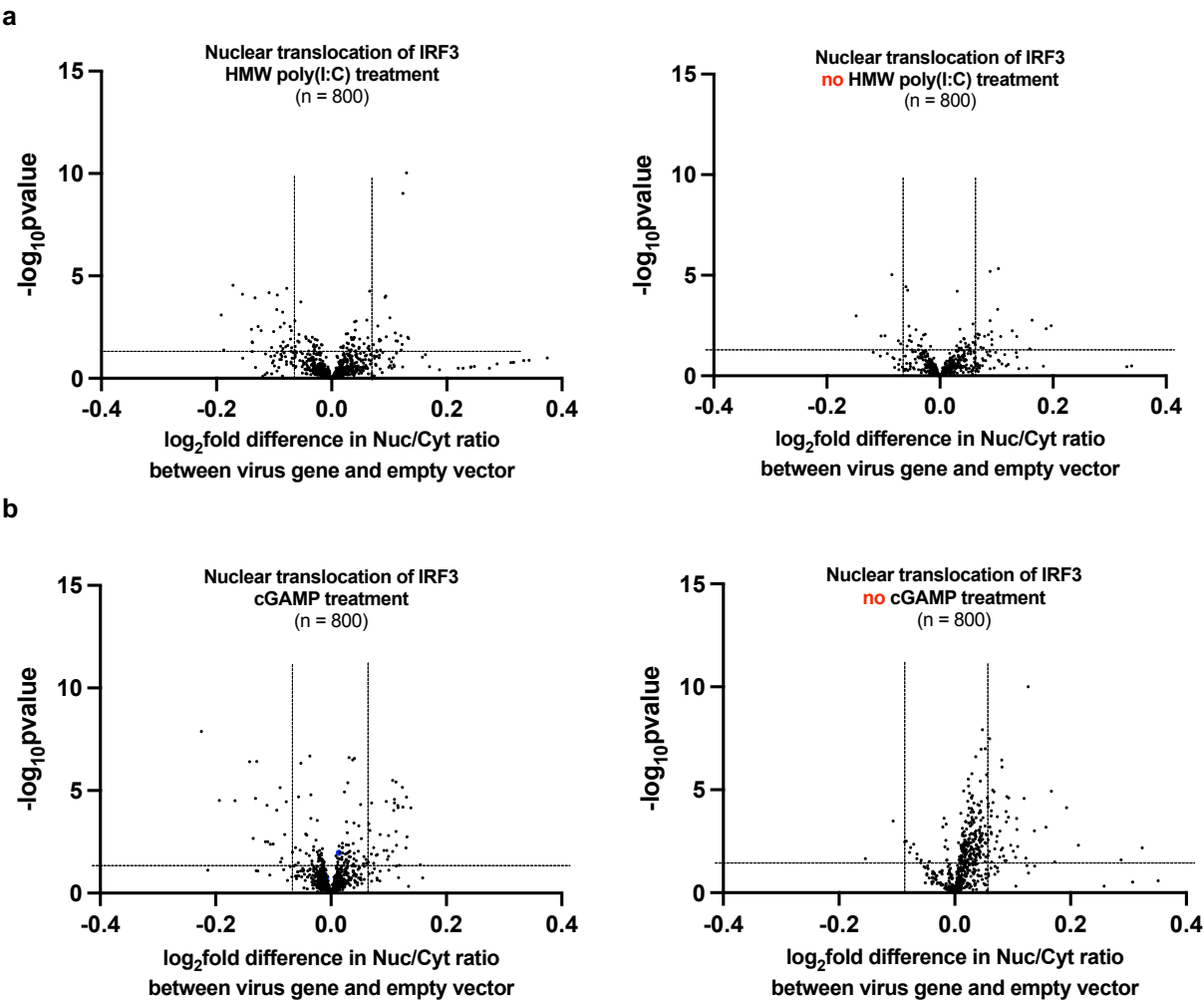

Supplement: FIG S2 [file msystems.01466-21-sf002.pdf]

Figure S3

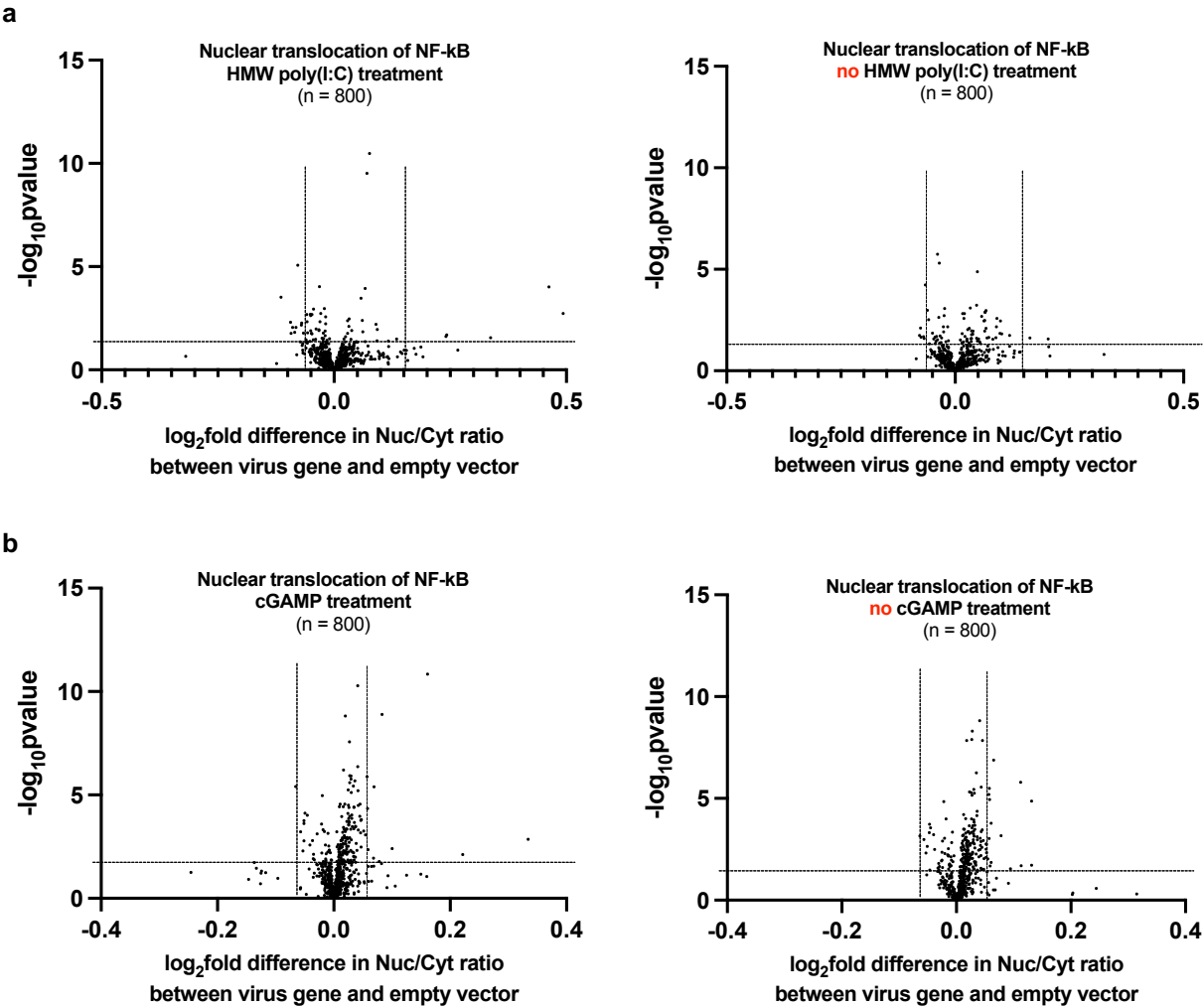

Supplement: FIG S3 [file msystems.01466-21-sf003.pdf]

Figure S4

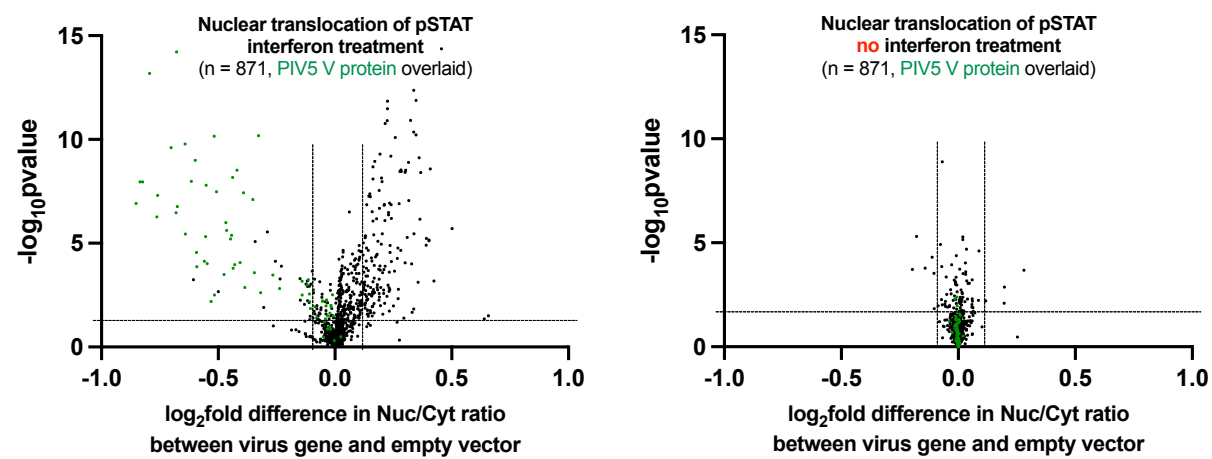

Supplement: FIG S4 [file msystems.01466-21-sf004.pdf]

Figure S6

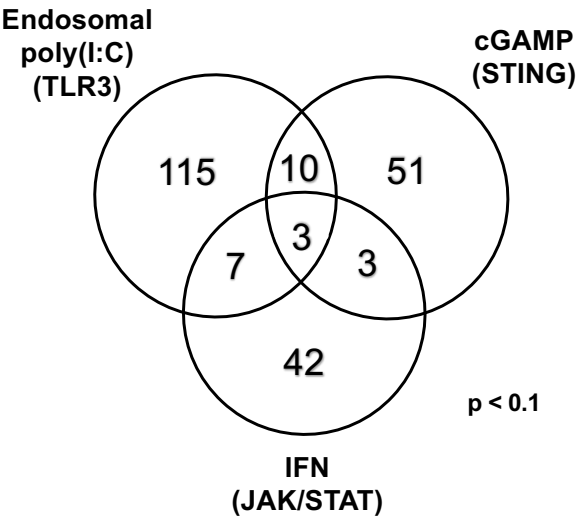

Supplement: FIG S6 [file msystems.01466-21-sf006.pdf]

Figure S7

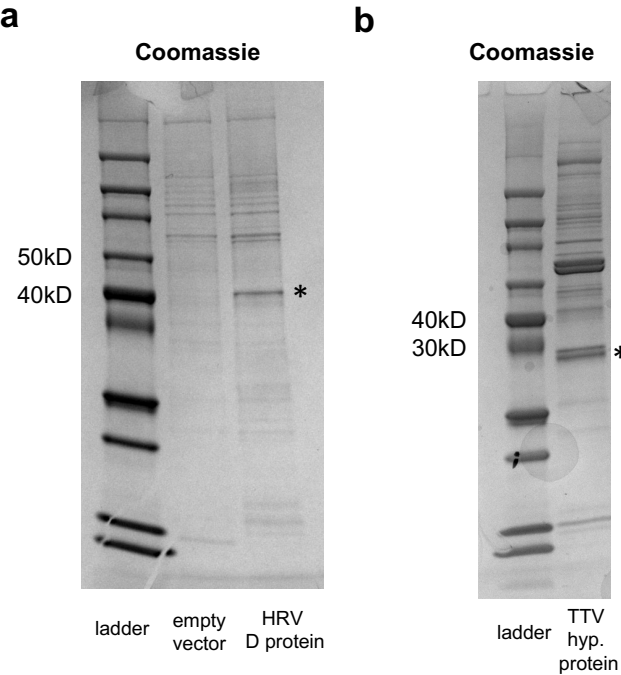

Supplement: FIG S7 [file msystems.01466-21-sf007.pdf]
