# Supplementary material for: High-Content Screening and Computational Prediction Reveal Viral Genes That Suppress the Innate Immune Response
Source: mSystems. 2022 Mar 23;7(2):e01466-21. doi: 10.1128/msystems.01466-21 (PMC9040872; doi:10.1128/msystems.01466-21)

Figure S5

a) Nuclear translocation of IRF3, cGAMP treatment

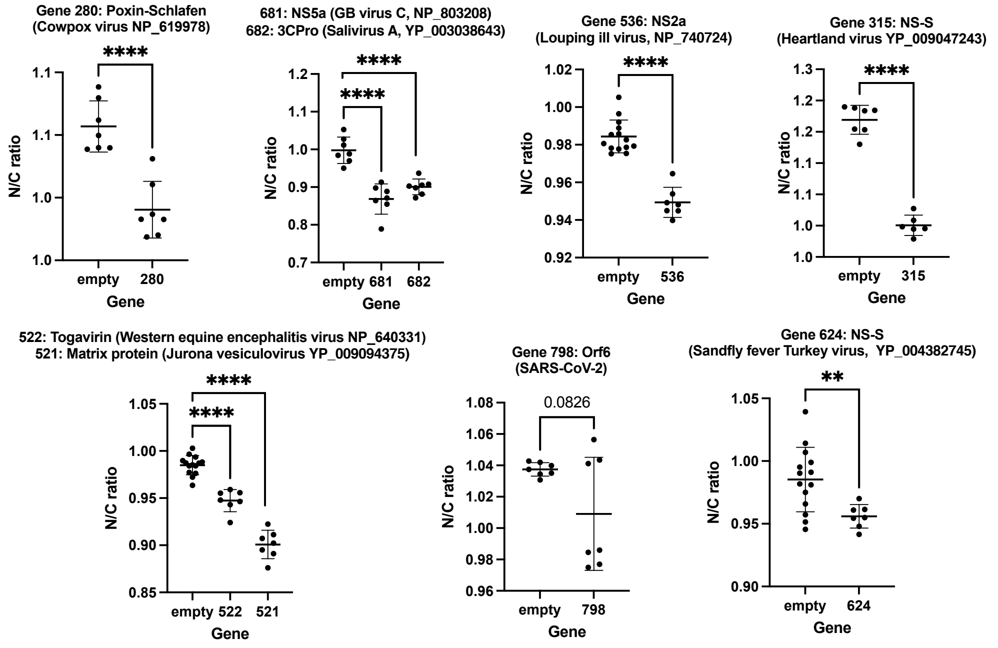

b) Nuclear translocation of IRF3, poly(I:C) treatment

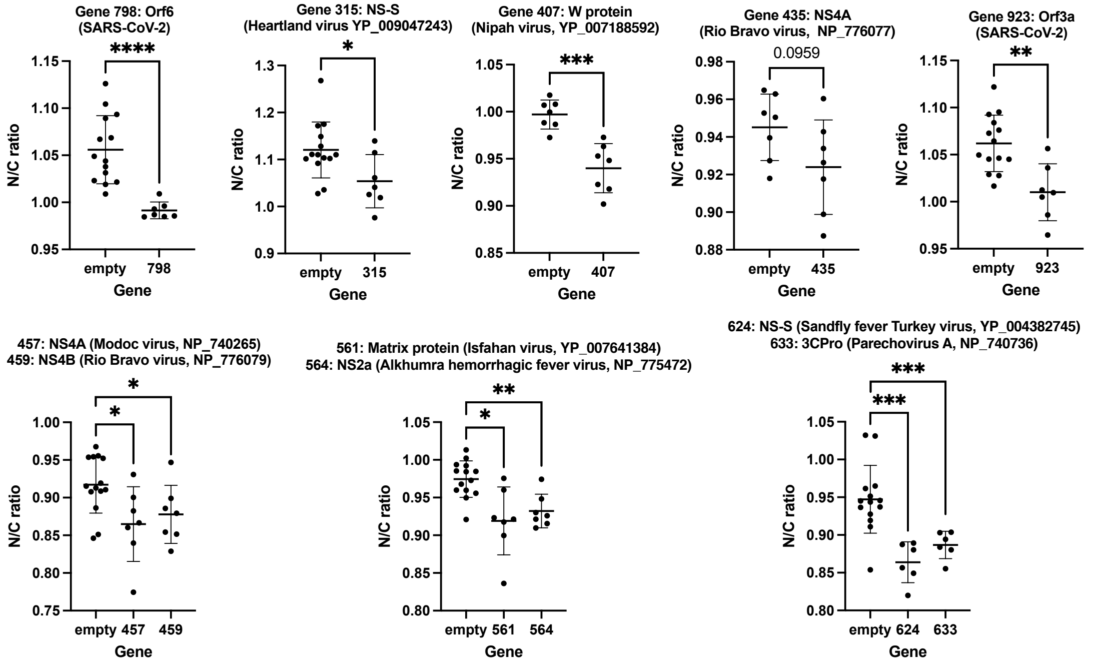

c) Nuclear translocation of NF-κB, cGAMP treatment

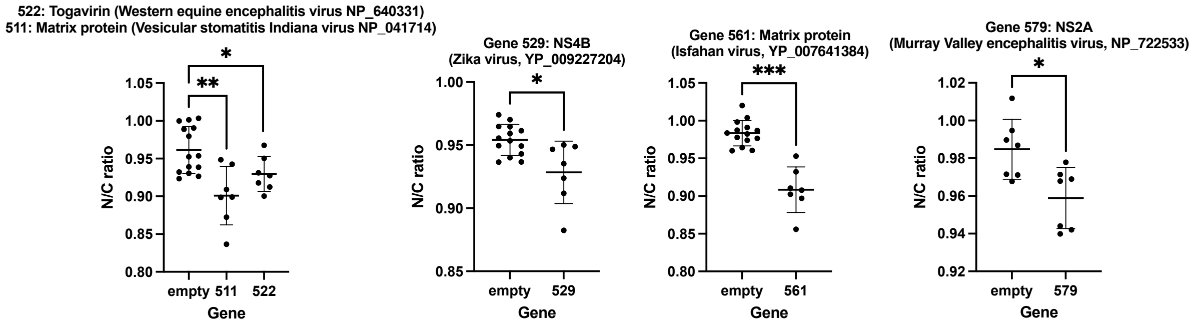

Supplement: FIG S5 [file msystems.01466-21-sf005.pdf]
